# Effects of *Lactobacillus plantarum* CJLP55 on Clinical Improvement, Skin Condition and Urine Bacterial Extracellular Vesicles in Patients with Acne Vulgaris: A Randomized, Double-Blind, Placebo-Controlled Study

**DOI:** 10.3390/nu13041368

**Published:** 2021-04-19

**Authors:** Mi-Ju Kim, Kun-Pyo Kim, Eunhye Choi, June-Hyuck Yim, Chunpil Choi, Hyun-Sun Yun, Hee-Yoon Ahn, Ji-Young Oh, Yunhi Cho

**Affiliations:** 1 Department of Medical Nutrition, Graduate School of East-West Medical Science, Kyung Hee University, Yongin-si 17104, Gyeongggi-do, Korea; miju0522@gmail.com (M.-J.K.); kunpyokim@gmail.com (K.-P.K.); eunhye960718@naver.com (E.C.); 2 Department of Dermatology, Kyung Hee University Medical Center, Seoul 02447, Korea; laurel02@nate.com; 3 Skyfeel Dermatologic Clinic, Seoul 06020, Korea; aag0001@naver.com; 4 CJ Foods R & D Center, CJ CheilJedang Corporation, Suwon-si 16495, Gyeongggi-do, Korea; hs.yun@cj.net (H.-S.Y.); hy.ahn@cj.net (H.-Y.A.); jy.oh@cj.net (J.-Y.O.)

**Keywords:** *Lactobacillus plantarum* CJLP55, acne vulgaris, sebum, hydration, urine bacterial extracellular vesicles

## Abstract

*Lactobacillus plantarum* CJLP55 has anti-pathogenic bacterial and anti-inflammatory activities in vitro. We investigated the dietary effect of CJLP55 supplement in patients with acne vulgaris, a prevalent inflammatory skin condition. Subjects ingested CJLP55 or placebo (*n* = 14 per group) supplements for 12 weeks in this double-blind, placebo-controlled randomized study. Acne lesion count and grade, skin sebum, hydration, pH and surface lipids were assessed. Metagenomic DNA analysis was performed on urine extracellular vesicles (EV), which indirectly reflect systemic bacterial flora. Compared to the placebo supplement, CJLP55 supplement improved acne lesion count and grade, decreased sebum triglycerides (TG), and increased hydration and ceramide 2, the major ceramide species that maintains the epidermal lipid barrier for hydration. In addition, CJLP55 supplement decreased the prevalence of *Proteobacteria* and increased *Firmicutes*, which were correlated with decreased TG, the major skin surface lipid of sebum origin. CJLP55 supplement further decreased the *Bacteroidetes*:*Firmicutes* ratio, a relevant marker of bacterial dysbiosis. No differences in skin pH, other skin surface lipids or urine bacterial EV phylum were noted between CJLP55 and placebo supplements. Dietary *Lactobacillus plantarum* CJLP55 was beneficial to clinical state, skin sebum, and hydration and urine bacterial EV phylum flora in patients with acne vulgaris.

## 1. Introduction

Acne vulgaris is characterized by excess sebum production and follicular hyperkeratinization [1,2]. Additionally, bacterial colonization with *Cutibacterium acnes* (*C. acnes*: formerly *Propionibacterium acnes* (*P. acnes*)), which uses sebum as the main nutrient to proliferate and releases proinflammatory cytokines, contributes to the development of acne vulgaris [1,2]; therefore, topical and systemic antibiotics, often in combination each other, have been a mainstay for the treatment of acne vulgaris [3,4]. However, the nonspecific chemical eradication of *C. acnes* coupled with the emergence of bacterial resistance has led to a new insight into the utilization of probiotics for skin health improvement [3,4].

Kimchi is a Korean traditional fermented food that is made of various vegetables such as Chinese cabbage, radish, garlic, ginger, and red pepper (chili). As a well-known healthy food in the world [5], kimchi contains various nutritional components such as vitamins, minerals, fiber, and phytochemicals. Moreover, nonpathogenic lactic acid producing bacteria (LAB), involved in the process of kimchi fermentation, are considered to be responsible for its health benefits [6]. *Lactobacillus*, the major LAB species in kimchi, modulates intestinal or systemic bacterial flora and systemically exerts anti-inflammatory effects that extend beyond the gut and may even affect the skin [7,8].

Of the various *Lactobacillus* strains isolated from kimchi, *Lactobacillus plantarum* (*L. plantarum*) CJLP55 tolerates low pH and a high bile salt concentration under gastrointestinal conditions [6], which is reflective of the stability and continuity of *L. plantarum* in the intestine [9]. *L. plantarum* CJLP55 has been reported to have immunomodulation effects with altered cytokine productions [10,11]. *L. plantarum* CJLP55 increases interleukin (IL)-12 levels and decreases IL-4 levels in ovalbumin-sensitized mouse splenocytes in vitro [10]. Orally administered *L. plantarum* CJLP55 decreases the production of IL-4 and IL-5 in lymph nodes and relieves atopic dermatitis (AD)-like skin lesions in NC/Nga mice [11]. In addition, ingested or topically applied *L. plantarum* modulates bacterial flora in the intestine as well as in the skin [12,13,14]. However, bacteria might not be disseminated in the body and barely reach directly to the skin [15]. Instead, extracellular vesicles (EV), the nanometer-sized vesicles released from bacteria, can move freely throughout body and indirectly affect the process of skin inflammation [15,16]. In fact, orally administered *L. plantarum* CJLP55-derived EV decreases IL-4 production and inflammation in the skin of an AD-like animal model [16], but little information is available on the systemic effect of *L. plantarum* CJLP55 on acne vulgaris, a prevalent inflammatory skin disease. In this randomized, double-blind, placebo-controlled pilot study, we investigated the dietary effect of *L. plantarum* CJLP55 supplement on clinical symptoms of acne vulgaris. In addition, we further determined its effects on skin condition-related factors, skin surface lipids, and urine bacterial extracellular vesicles (EV), a potential biomarker of the altered systemic bacterial flora [16], in patients with acne vulgaris.

## 2. Materials and Methods

### 2.1. Preparation of Probiotics and Placebo Products

*L. plantarum* CJLP55 (KCTC 11401BP, GenBank accession number GQ336971) was provided by CJ Foods R & D Center, CJ CheilJedang Corporation (Suwon, Republic of Korea). For administration, lyophilized *L. plantarum* CJLP55 at a dosage of 1.0 × 10^10^ colony-forming units (CFU) was mixed with maltodextrin and glucose anhydrocrystalline per one airtight alu-bag, and stored at 4 °C until administration. Placebo was prepared with a mixture of maltodextrin and glucose anhydrocrystalline only, of which the identical appearance and taste were confirmed by CJ Foods R & D Center. One alu bag of CJLP55 or placebo products provided 10 kcal and 2 g of carbohydrates.

### 2.2. Study Subjects

This study was approved by the Institutional Review Board of Kyung Hee University (Yongin, Republic of Korea) (KHSIRB 2015-013) and was conducted at Kyung Hee University Medical Center, Seoul, Korea in accordance with the Declaration of Helsinki. In addition, this study was registered at the Clinical Research Information Service (No. KCT0005401), a non-profit online registration system for clinical trials established by the Korea Centers for Disease Control and Prevention (KCDC). The KCDC joined the World Health Organization’s International Clinical Trials Registry Platform to become the 11th member of its Primary Registry, and the full trial protocol for this study is available there.

During July 2016, male and female patients with acne vulgaris were recruited. The inclusion criteria for this study were ages of 19–39 years, mild to moderate acne vulgaris and excess sebum on the face with normal body mass index (BMI) (18.5 to 24.9 kg/m^2^). Specifically, diagnosis for mild-to-moderate acne vulgaris on the face (defined as acne grade of ≥2.0 to <4.0 with at least 15 inflammatory and/or non-inflammatory lesions but no more than 3 nodules on the face) was based on the criteria scale of Investigator’s Global Assessment (IGA). Diagnosis for excess sebum on the face (defined as > 150 μg/cm^2^ sebum on the forehead) was based on the criteria scale of sebum content for oily skin by SM815 sebumeter [17]. Exclusion criteria were acne treatment of any kind including artificial ultraviolet (UV) therapy in the previous 2 months, concomitant other skin disease or systemic illnesses, use of medication that could interfere with acne, or failure to maintain normal BMI with a steady weight (± 4 kg) throughout the study. Written informed consent forms were obtained from each subject prior to enrollment of the study.

### 2.3. Study Design

This was a double-blind, placebo-controlled, parallel-group clinical study. Based on our previous study [18], a sample size of 9 per group was determined to provide > 80% power at a 2-tailed α = 0.05 for a difference of 62% with a SD of 33% in levels of triglycerides (TG), a major skin surface lipid [19]. In consideration of a 10% dropout rate and 70% compliance, we aimed to recruit 15 subjects per group. For the current study 30 subjects were randomly assigned to either the CJLP55 (*n* = 15, 7 males and 8 females) or the placebo (*n* = 15, 5 males and 10 females) groups (Figure S1). Upon confirmation of the inclusion and exclusion criteria, randomization was performed by a researcher who was not involved in this study. Eligible subjects were randomly allocated into the two arms of the study in a 1:1 ratio, according to randomly permuted blocks within the strata of two assignments, the CJLP55 and placebo groups. The allocation sequence was not available to the subjects, the dermatologist, or any of the researchers involved in this study.

Subjects were instructed to ingest one bag of placebo product (placebo group) or 1.0 × 10^10^ CFU of *L. plantarum* CJLP55 (CJLP55 group) daily for 12 weeks from July 2015 to October 2015. Subjects were also instructed to refrain from consuming any other probiotic products 2 weeks before the beginning of the study and until the study had finished. In addition, to avoid the influence of other factors on skin, subjects were instructed to maintain their usual cleaning habits and moisturizing products throughout the study. However, to ensure the accuracy of the corneometer, sebumeter, and skin-pH meter, subjects were instructed to avoid use of any moisturizing products on the days of dermatological assessment. During the 12-week study period, compliance was monitored weekly via regular telephone interviews and all adverse events, including gastrointestinal discomfort, were recorded. Anthropometry measures, blood samples for hematology and biochemistry analyses, and a routine urine analysis with a pregnancy test (females only) were performed at 0 week (baseline) and 12 weeks. The primary outcome of this study was clinical assessment of facial acne with the acne-related lesion count and acne severity. All other measures were secondary outcomes.

### 2.4. Clinical Assessment of Facial Acne

Photographs of the facial area were taken under standardized conditions at baseline and 12 weeks. Clinical assessment of facial acne was performed at baseline and 12 weeks by the same dermatologist who was blind to the group assignment of subjects. The dermatologist counted acne-related lesions (noninflammatory lesions: open and closed comedones; inflammatory lesions: papule, pustule and nodule) and evaluated acne severity according to IGA scale at baseline and 12 weeks.

### 2.5. Measurement of Skin Condition-Related Factors

Under the standardized room conditions of 22–24 °C and 55–60% humidity, subjects were given at least 30 min to relax so that the skin condition could equilibrate. Skin sebum, hydration, and pH of forehead were measured using sebumeter (SM815), corneometer (CM825), and skin pH-meter (PH905) (all from Courage-Khazaka, Cologne, Germany) at baseline and 12 weeks, as described previously [18]. Skin sebum, hydration and pH values were the average of 5 determinations after equilibrium was attained. The temperature and humidity of the probe were also recorded.

### 2.6. Analysis of Skin Surface Lipids

The skin surface of the forehead was stripped using 10 tape strips (14 mm D-SQUAME^®^ Tape; Cu-Derm Corporation, Dallas, TX, USA) at baseline and 12 weeks. All tape strips from each subject were combined and stored at −20 °C until further processing. After corneocytes were removed from the tape by sonication in methanol, the lipids were extracted and fractionated by high performance thin layer chromatography (HPTLC), as descried previously [18]. The fractions containing TG, free fatty acids (FFA), cholesterol (Chol), total ceramides (Cer), and ceramide 2 (Cer2) that comigrated with respective standards were scanned at 420 nm with a TLC III scanner (CAMAG, Muttenz, Switzerland) and were quantified by calibration curves using various concentrations of external standards of each lipid. Levels of total skin surface lipids including TG, FFA, Chol, total Cer, and Cer2 were expressed as ng/μg protein.

### 2.7. Metagenomic DNA Analysis of Bacterial EV Phylum in Urine

As an indirect method to analyze the alteration of systemic bacterial flora [16], metagenomic analysis of bacterial DNA was performed in urine EV [20]. Urine samples from subjects who gave written informed consent for urine sample collection (*n* = 9 in the CJLP55 group, 4 males and 5 females; *n* = 12 in placebo group, 4 males and 8 females) were collected at baseline and 12 weeks. EV were purified from urine by multiple ultracentrifugations, and sequentially, bacterial DNA was extracted according to the manufacturer’s instructions (PowerSoil DNA Isolation Kit; MO Bio, Carlsbad, CA, USA), as described previously [20,21]. The extracted bacterial DNA was quantified by the QIAxpert system (QIAGEN, Hilden, Germany). The polymerase chain reaction (PCR) for variable 3 (V3) and variable 4 (V4) regions of 16S ribosomal RNA (rRNA) genes, which can be sequenced to discriminate the bacterial phylogenetic identification [15], was performed using the primer set of 16S_V3 forward primer (5’-TCGTCGGCAGCGTCAGA

TGTGTATAAGAGACAGCCTACGGGNGGCWGCAG-3′) (50mer) and 16S_V4 reverse primer (5′-GTCTCGTGGGCTCGGAGATGTGTATAAGAGACAGGACTACHVGGGTATCTAATCC-3′) (55 mer) [20,21]. The PCR products were used for the construction of 16S ribosomal DNA gene libraries following the MiSeq System guidelines (Illumina Inc., San Diego, CA, USA). The 16S rRNA gene libraries for each sample were quantified using QIAxpert (QIAGEN, Hilden, Germany), pooled at the equimolar ratio, and used for pyrosequencing with the MiSeq System (Illumina Inc., San Diego, CA, USA) according to the manufacturer’s recommendations. The operational taxonomy unit (OTU) was analyzed using UCLUST and USEARCH, with phylogenetic classification performed using QIIME based on the 16sRNA sequence database of GreenGenes 8.15.13. Based on >75% similarity, all sequences were classified for phylum profile. The proportion of each bacterial EV phylum was evaluated as a percentage of the total phylum from the sequence reads, and the *Bacteroidetes*:*Firmicutes* (B/F) ratio was then determined.

### 2.8. Statistics

All statistical analyses were carried out on a per-protocol basis with SPSS 21.0 for Windows (SPSS Inc., Chicago, IL, USA). Values are presented as mean ± SEM. Differences from baseline within each group were analyzed by paired Student’s *t* test for normally distributed variables and Wilcoxon’s signed rank test for non-normally distributed variables. Differences between groups were analyzed by unpaired Student’s *t* test for normally distributed variables and Mann–Whitney *U* test for non-normally distributed variables. Raw data are available as Supplementary Materials (File S1: Raw research data).

The difference in observed numbers of male and female subjects from theoretical frequencies between groups was analyzed by a chi-square test (Table 1). Univariate analysis was performed to determine the influence of sex on percent changes of acne lesion count and acne grade over 12 weeks using a generalized linear model for inflammatory lesion count (ILC) or generalized linear mixed model for total lesion count (TLC) and acne grade (Table 2). In this analysis, acne lesion count or acne grade was used as a dependent variable and sex (females coded as 0; males coded as 1) was used as an independent variable. Principle component analysis (PCA) was performed to determine a separation of percent changes of bacterial EV phylum profiles over 12 weeks between groups. Pearson’s correlation analysis was performed to determine correlations of percent changes of TG levels with those of the phyla *Proteobacteria* or *Firmicutes* proportions over 12 weeks in subjects of the CJLP55 group. Two-sided *p* values <0.05 were considered significant.

## 3. Results

### 3.1. Study Subjects

Initially, 30 subjects were randomly assigned to either the CJLP55 (*n* = 15, 7 males and 8 females) or the placebo (*n* = 15, 5 males and 10 females) groups (Figure S1). During the 12-week study period, one female in the CJLP55 group dropped out for personal reasons, and one female in the placebo group who failed to maintain a steady weight and became underweight (BMI < 18.5) was excluded from analyses. Therefore, data were analyzed for 14 subjects (7 males and 7 females) in the CJLP55 group and 14 subjects (5 males and 9 females) in the placebo group (File S2: Consort 2010 checklist).

Enrolled numbers of male and female subjects (5 males and 9 females) in the placebo group were not significantly different from theoretical frequencies (7 males and 7 females) (*p* = 0.445) (Table 1). There were no statistical differences in ages and BMI between groups. The acne grade at baseline matched well to dermatologic assessment of ILC and TLC, all of which were not significantly different between groups. Tolerability of CJLP55 and placebo products was excellent in both groups. Changes of usual cleaning and moisturizing products and adverse events including gastrointestinal discomfort were not reported during the study period. Hematology, serum, and urine analyses of all subjects were normal at baseline and 12 weeks.

### 3.2. Clinical Assessment of Facial Acne

Compared to baseline, ILC, TLC, and acne grade for male and female subjects in the CJLP55 group were significantly decreased at 12 weeks; therefore, acne lesion counts and acne grade for all subjects (male and female combined) were significantly decreased at 12 weeks in the CJLP55 group (Table 2). In placebo group, ILC, TLC, and acne grade for male and female subjects remained unchanged, but ILC for all subjects was significantly decreased during the 12-week study period.

When the differences in percent changes over 12 weeks were compared between groups, the percent change of ILC for male and female subjects between CJLP55 and placebo groups was not significantly different, but it was significantly decreased by 42.09% (95% confidence interval (CI), −80.57 to −3.61; *p* = 0.033) for all subjects in the CJLP55 group compared with the placebo group. The TLC was significantly decreased by 41.38% (95% CI, −73.67 to −9.10; *p* = 0.017) for male, 45.51% (95% CI, −94.67 to 3.65; *p* = 0.031) for female, and 49.67% (95% CI, −82.76 to −16.59; *p* = 0.002) for all subjects in the CJLP55 group. In parallel, the acne grade in the CJLP55 group was also decreased significantly by 29.33% (95% CI, −51.36 to −7.29 to; *p* = 0.030) for male, 29.89% (95% CI, −54.04 to −5.74; *p* = 0.012) for female, and 28.99% (95% CI, −44.23 to −13.75; *p* = 0.009) for all subjects, compared with the placebo group.

**Figure 1:**
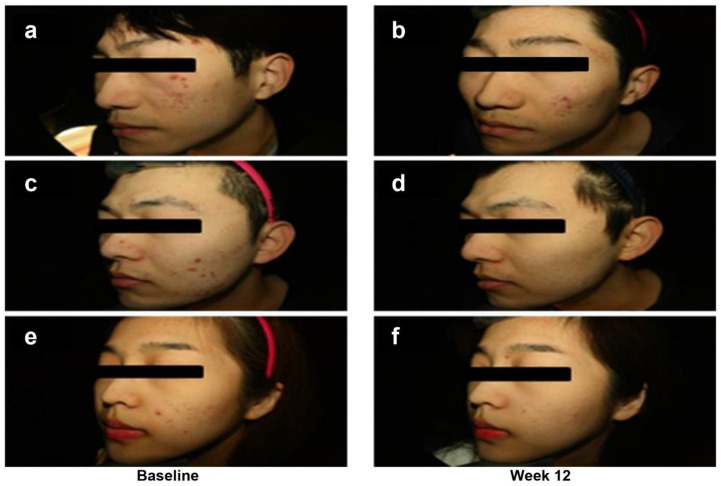
Photographs of acne improvement in subjects A (**a**,**b**), B (**c**,**d**), and C (**e**,**f**) in the CJLP55 group at baseline and week 12, respectively.

Despite sex imbalance in the placebo group (*n* = 14, 5 males and 9 females), sex was not a significant discriminating factor for the percent changes of acne lesion count and acne grade over 12 weeks (CJLP55 group: *p* = 0.539 in ILC, *p* = 0.244 in TLC, *p* = 0.383 in acne grade; placebo group: *p* = 0.917 in ILC, *p* = 0.699 in TLC, *p* = 1.000 in acne grade) in each group. Representative photographs of acne improvement in the CJLP55 group are shown in Figure 1.

### 3.3. Measurement of Skin Condition-Related Factors

There was no sex effect on acne lesion counts and acne grade. Therefore, the data of male and female subjects for skin condition were combined as all subjects (Table 3). Compared to baseline, the skin pH of the CJLP55 and placebo groups remained unchanged at 12 weeks, with no difference in percent change between these two groups. Moreover, skin hydration of these two groups remained unchanged at 12 weeks compared to baseline; however, the positive percent change of the CJLP55 group and the negative percent change of the placebo group over 12 weeks resulted in a significant difference between these two groups. Therefore, the skin hydration in the CJLP55 group was increased by 14.52% (95% CI, 6.47 to 22.57; *p* = 0.003) compared to the placebo group. Notably, sebum content of the CJLP55 group was significantly decreased whereas that of the placebo group remained unchanged over 12 weeks, resulting in a 16.27% decrease (95% CI, −32.28 to −0.26; *p* = 0.005) of sebum content in the CJLP55 group, compared to the placebo group.

### 3.4. Analysis of Skin Surface Lipids

**Figure 2:**
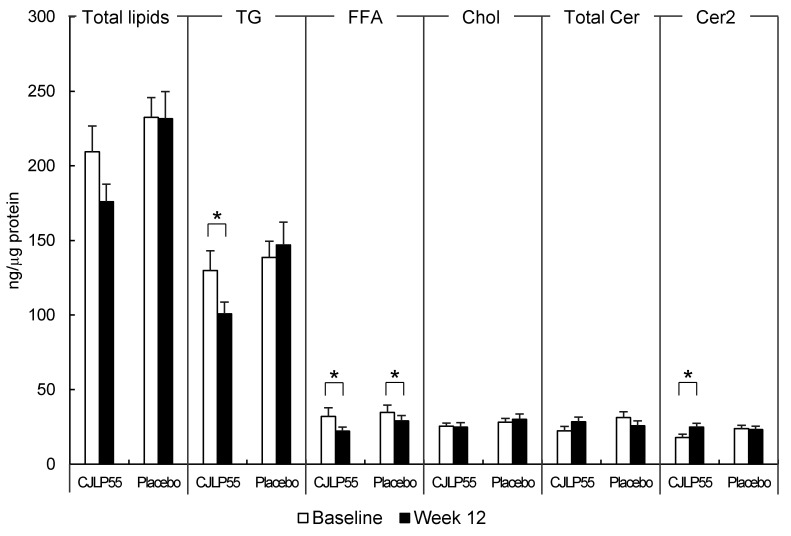
Altered levels of skin surface lipids in CJLP55 and placebo groups. Values are mean ± SEM (CJLP55 group: *n* = 14; placebo group: *n* = 14). Differences between baseline (white bars) and week 12 (black bars) in lipid fractions of the CJLP55 or placebo groups were evaluated using paired *t*-test test (Total lipids, Chol, Cer2) or Wilcoxon signed rank test (TG, FFA, Total Cer) (* *p* < 0.05). TG, triglycerides; FFA, free fatty acids; Chol, cholesterol; Cer, ceramides; Cer2, ceramide 2.

In HPTLC analysis, the level of total skin surface lipids in CJLP55 and placebo groups remained unchanged over 12 weeks (CJLP55 group: 209.46 ± 17.30 ng/μg protein at baseline, 175.97 ± 11.66 ng/μg protein at 12 weeks; placebo group: 232.52 ± 13.24 ng/μg protein at baseline, 231.64 ± 18.13 ng/μg protein at 12 weeks) (Figure 2), which was not consistent with the decreased sebum content in the CJLP55 group over 12 weeks (Table 3). Further fractionation of total skin surface lipids revealed that TG, FFA, Chol, total Cer and Cer2 were separated. TG were the most abundant and comprised > 57% of the total skin surface lipids of the forehead in subjects with acne in the CJLP55 and placebo groups, as reported previously [19]. FFA, Chol, and total Cer including Cer2 and other Cer species accounted for < 17% of total skin surface lipids. Levels of TG and FFA were significantly decreased in the CJLP55 group whereas the level of FFA only was significantly decreased in the placebo group over 12 weeks: The level of TG remained unchanged in the placebo group over 12 weeks, thereby resulting in a 28.44% decrease of TG specifically (95% CI, −54.08 to −2.79; *p* = 0.031), but not FFA, in the CJLP55 group compared to the placebo group. Although the level of total Cer remained unchanged in CJLP55 and placebo groups, the level of Cer2, the major Cer species of human epidermis [22], was increased over 12 weeks in the CJLP55 group. Other Cer species were barely detectable with low band intensities, of which alterations between CJLP55 and placebo groups were not apparent.

### 3.5. Metagenomic DNA Analysis of Bacterial EV Phylum in Urine

**Figure 3:**
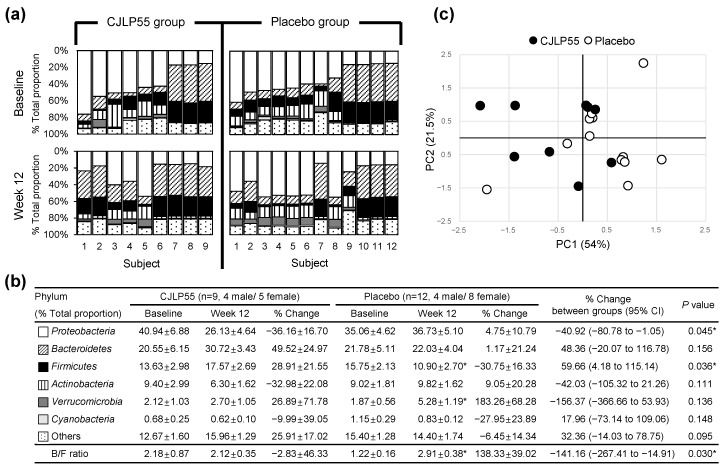
Altered abundance of bacterial extracellular vesicle (EV) phyla in urine of CJLP55 and placebo groups. (**a**) Altered % total proportion of urine bacterial EV phyla from baseline to 12 weeks in individual subjects of groups. The horizontal axis displays the subject number. (**b**) Altered % total proportion of urine bacterial EV phyla over 12 weeks in groups. Phyla present at less than 0.5% are noted as “others.” B/F ratio: *Bacteroidetes* to *Firmicutes* ratio. All values in (**b**) are mean ± SEM (CJLP55 group: n = 9; placebo group: n = 12). Differences from baseline within CJLP55 or placebo groups in (**b**) were determined by Wilcoxon signed rank test Differences of percent change between CJLP55 and placebo groups in (**b**) were determined by unpaired Student’s *t* test (*Proteobacteria*, *Bacteroidetes*, *Firmicutes*, *Verrucomicrobia*, *B/F ratio*) or Mann–Whitney *U* test (*Actinobacteria*, *Cyanobacteria*, *Others*) (* *p* < 0.05). (**c**) Principle component analysis (PCA) score on percent changes of bacterial EV phyla over 12 weeks in groups.

EV, the nanometer-sized vesicles released from archaea, bacterial, and eukaryotic cells, have been observed in body fluids such as serum and urine [15,16,20,21]. DNA phylum profiles of bacterial origin are similar in EV from either serum or urine [16]. Therefore, metagenomic analysis of urine bacterial EV can indirectly reflect the alteration of systemic bacterial flora. When DNA phylum profiles of urine bacterial EV were analyzed at levels of >0.5% in individual subjects of CJLP55 and placebo groups, the phyla *Proteobacteria*, *Bacteroidetes*, *Firmicutes*, *Actinobacteria*, *Verrucomicrobia*, and *Cyanobacteria* (in order from high to low proportions) were present. When the mean values of each phylum were assessed as percent of total proportion, *Proteobacteria* was the predominant phylum, comprising >35% of all bacteria, and *Cyanobacteria* was the least present at baseline of these two groups (Figure 3a,b).

Compared to baseline, the proportion of *Proteobacteria* remained unchanged at 12 weeks in the CJLP55 and placebo groups, but the negative percent change of the CJLP55 group and the positive percent change of the placebo group over 12 weeks resulted in a significant difference between these two groups. The proportion of *Firmicutes* remained unchanged in the CJLP55 group, but it was decreased in the placebo group at 12 weeks; these alterations resulted in a 40.92% decrease (95% CI, −80.78 to −1.05; *p* = 0.045) of *Proteobacteria* and a 59.66% increase (95% CI, 4.18 to 115.14; *p* = 0.036) of *Firmicutes* over 12 weeks in the CJLP55 group compared to the placebo group (Figure 3a,b). Compared to baseline, the proportion of *Verrucomicrobia* was significantly increased in the placebo group; however, its similar trend noted at 12 weeks in the CJLP55 group resulted in no difference of percent change over 12 weeks between these two groups. The proportion of other phyla, including *Bacteroidetes, Actinobacteria*, *Cyanobacteria* and others (phyla present at less than 0.5%), remained unchanged over 12 weeks in the CJLP55 and placebo groups, thereby resulting in no difference in percent change of these phyla over 12 weeks between these two groups. However, despite no altered proportion of *Bacteroidetes* over 12 weeks, decreased proportion of *Firmicutes* induced a shift toward a significant increased *Bacteroidetes*:*Firmicutes* (B/F) ratio, a relevant biomarker of bacterial dysbiosis [16], in the placebo group, whereas its ratio remained unchanged in the CJLP55 group with no altered proportion of these two phyla over 12 weeks, therefore resulting in a 141.16% decrease (95% CI, −267.41 to −14.91; *p* = 0.030) of B/F ratio over 12 weeks in the CJLP55 group compared to the placebo group. The dysbiosis of urine bacterial EV phylum in the placebo group was further evident by PCA analysis. PCA scores for the CJLP55 group (filled circle in Figure 3c) were relatively separated from those for the placebo group (open circle in Figure 3c), which implies that there is a distinction of percent changes of urine bacterial EV phylum profiles between CJLP55 and placebo groups.

### 3.6. Correlations between Percent Changes in TG Levels and in Proteobacteria and Firmicutes Proportions

**Figure 4:**
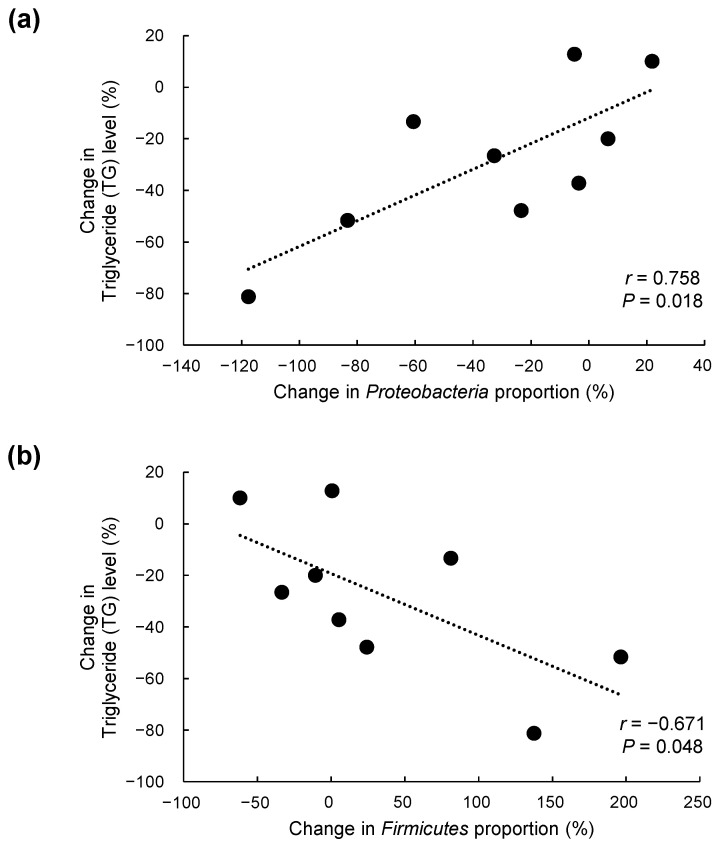
Correlations between percent changes in triglyceride levels and in (**a**) *Proteobacteria* and (**b**) *Firmicutes* proportions over 12 weeks in individual subjects of the CJLP55 group (*n* = 9).

The level of TG, the major skin surface lipid, was selectively decreased in the CJLP55 group over 12 weeks. In parallel, compared to the placebo group, the percent changes of *Proteobacteria* and *Firmicutes* were significantly altered in the CJLP55 group over 12 weeks. Therefore, the correlations between percent changes in TG level and those in the phyla *Proteobacteria* and *Firmicutes* proportions over 12 weeks were further determined in individual subjects of the CJLP55 group. The decreased percent change of TG level was positively correlated with that of the *Proteobacteria* proportion (*r* = 0.758, *p* = 0.018), whereas it was negatively correlated with increased percent change of the *Firmicutes* proportion (*r* = −0.671, *p* = 0.048) (Figure 4). These results indicate that the selectively decreased TG level and decreased *Proteobacteria* with a concomitant increase in *Firmicutes* in urine were metabolic features that led to decreases in sebum content and acne lesion count, ultimately improving acne grade in the CJLP55 group.

## 4. Discussion

*Lactobacillus*, a well-characterized genus in LAB, is beneficial for skin health improvement [7,8]. Specifically, various strains of *L. plantarum* have been reported to improve acne vulgaris with broad anti-inflammatory and anti-pathogenic bacterial activities as well as with modulation of bacterial flora in the skin [12,14,23]. Topically applied *L. plantarum* on the face of patients with mild acne vulgaris reduces acne lesion size and erythema [12]. Treatment with *L. plantarum* THG-10 in vitro decreases the growth of *C. acnes* and nitric oxide production, which is induced by the inflammation process [23]. Furthermore, results from topically applied studies in female subjects indicate that *L. plantarum*-GMNL6 reduces erythema and the proportion of *Propionibacterium* genus in the face [14]. In this study, we demonstrated that *L. plantarum* CJLP55 supplement decreased the clinical severity of acne. Compared to baseline, ILC, TLC, and acne grade were significantly decreased in the CJLP55 group over 12 weeks. For the placebo group, ILC was decreased, but TLC and acne grade remained unchanged over the 12 weeks.

In addition to the decreased clinical severity of acne, sebum content also decreased and skin hydration improved in the CJLP55 group, but the levels of FFA significantly decreased during the 12 weeks for both the CJLP55 and placebo groups. During this period, a decrease in ILC was also detected for both groups, suggesting that the decreases in FFA and ILC might be caused by ingestion of maltodextrin and glucose anhydrocrystalline. These are the common vehicle in the CJLP55 and placebo products. High intake of hyperglycemic carbohydrates is known to aggravate acne based on the hyperinsulinemia and androgen stimulated sebum production [24]. Maltodextrin and glucose anhydrocrystalline consist of pure glucose molecules. Since the supplemented amounts of these in the CJLP55 and placebo products are low, only 2 g carbohydrate (10 kcal), the glycemic load will be very low. They are therefore not likely to affect the insulin levels. Further studies are required to determine the effects of small but long-term carbohydrate intake on ILC and FFA of skin surface lipids in acne patients. TG is the major sebum lipid [19], whereas Cer2 is the major Cer species that maintains lamellar integrity of the epidermal barrier against water loss [22]. Notably, the levels of TG decreased but the level of Cer2 increased during the 12 weeks for the CJLP55 group. These results, coupled with the reports of increased sebum production and impaired epidermal barrier with reduced Cer levels in acne patients [25], indicate that *L. plantarum* CJLP55 supplement improved acne vulgaris with selectively decreased TG of sebum, and increased Cer2 and skin hydration.

The beneficial effects of *L. plantarum* CJLP55 supplement in the skin were further accompanied by altered bacterial EV phylum in urine of the CJLP55 group. In addition to providing diverse flora in the intestine, bacteria produce EV, which have been observed in the blood and urine [16,26,27]. Over 12 weeks, there was a percent decrease of *Proteobacteria* proportion in the CJLP55 group compared to the placebo group. Since not all bacteria produce EV [28], bacterial EV phylum flora in urine could be different from intestinal or skin bacterial flora, but the majority of acne patients have an unusual presence of several genera including *Klebsiella* and *Enterobacter*, which are affiliated with phylum *Proteobacteria* in intestinal bacterial flora [29]. Furthermore, a decreased proportion of *Bifidobacterium* and *Lactobacillus*, two major beneficial anaerobic bacterial genera [30], as well as an increased proportion of *Staphylococcus aureus* (*S*. *aureus*), are frequently reported in intestinal or skin bacterial flora of acne patients [25,29]. There was a percent increase of *Firmicutes*, with which *Lactobacillus* genus is affiliated [30], over 12 weeks in the CJLP55 group compared to the placebo group, but the pathogenic bacteria species of *S*. *aureus* is also affiliated with phylum *Firmicutes* [29]. On the other hand, *Actinobacteria*, with which *Bifidobacterium* genus is affiliated [30], remained unchanged over 12 weeks in the CJLP55 group. In fact, no significant alterations of bacterial EV flora at the identified genus or species levels were observed over 12 weeks between the CJLP55 and placebo groups. Urine samples were obtained from a limited number of subjects, which might explain no alteration of bacterial EV genus or species profiles in this study. Modulation of bacterial EV profiles should be analyzed in depth with a larger number of subjects in future studies. However, compared to the place group, there was a significant decrease in the B/F ratio over 12 weeks in the CJLP55 group, and PCA analysis further confirmed a distinction in the percent changes of urine bacterial EV phylum profiles over 12 weeks between CJLP55 and placebo groups. Moreover, the decreased percent changes of TG levels were correlated with those of *Proteobacteria* proportions and a concomitantly increased percent change of *Firmicutes* proportion in urine EV of the CJLP55 group. Recent studies reported that *Proteobacteria* is predominant in the urine EV phylum of pediatric patients with AD, whereas *Firmicutes* is predominant in that of normal subjects [16], and the B/F ratio is higher in acne patients than in healthy controls [31]. Taken together, these results suggest that ingested *L. plantarum* CJLP55 is likely to restore the bacterial EV phylum profiles, specifically decreased *Proteobacteria* and increased *Firmicutes* proportions systemically, which are altered in inflammatory skin diseases such as AD or acne [16,29].

Regarding dietary or supplement intervention for acne improvement, insulin or insulin-like growth factor-1 (IGF-1)-induced activation of the phosphatidylinositol 3-kinase (PI3K)/Akt/mechanistic target of rapamycin complex 1 (mTORC1) (previously referred as mammalian target of rapamycin complex 1) signaling pathway has been recognized to play a fundamental role in the regulation of sebocyte lipogenesis and proliferation [32,33]. High intake of hyperglycemic carbohydrates as well as milk or enriched branched-chain amino acids (BCAA) induces postprandial rises of insulin and/or IGF-1 levels in serum [32,33]. As a well-established factor in acne pathogenesis, IFG-1 increases the mRNA and protein expressions of sterol response element binding protein-1 (SREBP-1), the key lipogenic factor which is the down-stream target of PI3K/Akt/mTORC1 signaling in sebocytes [32,33,34]. Therefore, low glycemic load in the diet has been recommended for clinical improvement and reduced sebum and inflammation in acne patients [24,35]. Notably, probiotic supplement intervention with *L. plantarum* Ln4 also decreases insulin and IGF-1 expression in the adipose tissue of mice [36] and pooled probiotic supplementation with *L. rhamnosus*, *L. acidophilus*, and *Bifidobacterium bifidumi* improves insulin sensitivity in the adipose tissue of mice, in parallel with the modulation of intestinal bacterial flora [37]. Together with these prior studies [36,37], the beneficial effect of *L. plantarum* CJLP55 supplement intervention on the skin of acne patients in this study could be explained by the inhibited effect on IFG-1-induced activation of PI3K/Akt/mTORC1 signaling. In fact, *L. rhamnosus* SP1 supplement decreases clinical severity and IGF-1 mRNA expression in the skin of acne patients [38]. However, the beneficial effects of various strains of *L. plantarum* on decreased acne severity and sebum production have been reported mostly from topically applied and in vitro studies [12,14,23], and little information is available on the relationship between ingested *L. plantarum* and altered levels of insulin, IFG-1 and BCAA in either the serum or skin of acne patients. Alternatively, *L. plantarum* CJLP55 has been reported to have immunomodulation effects with altered cytokine production [10,11]. When coupled with prior studies reported that PI3K/Akt/mTORC1 signaling pathway is induced by specific receptors of various stimuli including insulin and IGF-1 [32,33] as well as cytokines [39,40,41], these results suggest that after *L. plantarum* CJLP55 is ingested, systemic immunomodulation with altered cytokine production is likely to occur, ultimately inducing beneficial effects on skin such as the selectively decreased TG of sebum and increased Cer2 and skin hydration.

To explain the beneficial effect of ingested probiotics for skin health improvement, modulation of intestinal bacterial flora and their released metabolites with increased intestinal permeability were adopted for systemic immunomodulation based on gut-brain-skin axis [42]. Alternatively, from recent studies reported that bacteria produce EV, which can move freely throughout body, bacterial EV in serum or urine is implicated as a systemic bacterial factor for immunomodulation, such as altering cytokine levels [16,26,27]. Similar to orally administrated *L. plantarum* CJLP55, which decreases the production of T-helper (Th)-2 cytokines of adaptive immunity, such as IL-4 and IL-5 in lymph nodes and relieves AD-like skin lesions in NC/Nga mice [11], oral administration of *L. plantarum* CJLP55-derived EV for 25 days decreases IL-4 production and inflammation in the skin of an AD-like animal model [16]. Oral administration of *L. plantarum* CJLP133, another *L. plantarum* strain of kimchi, decreases IL-4 level and inversely increases interferon (IFN)-γ levels in the serum of NC/Nga mice [43]. Moreover, decreased lipogenesis of ingested dietary or plant alternatives with anti-acne efficacy is frequently accompanied by systemic alteration of adaptive immunity cytokines, such as IL-4, IL-10, IL-12, and IFN-γ individually or in combination [44,45,46]. In our previous studies [47], individual treatment of lower levels of Th-2 cytokines, such as 0.5×IL-4 (0.5 fold (×) IL-4 concentration based on its normal serum concentration (1.0×) and 0.5×IL-10, and higher level of Th-1 cytokine such as 5.0×IFN-γ (5.0 fold (×) IFN-γ concentration based on its normal serum concentration (1.0×)) decreases lipid content in human sebocytes, and this effect is greater with their combined treatment. Furthermore, in the context of inhibited PI3K/Akt/mTORC1 signaling with lower levels of IL-4 and IL-10 and/or chronic treatment of IFN-γ [39,40,41], the individual treatment of 0.5xIL-10 and the combined treatment of 0.5xIL-4, 0.5xIL-10 and 5.0xIFN-γ in human sebocytes decrease the protein expression of mature SREBP-1 and/or fatty acid synthase (FAS) [47], of which expression is upregulated by SREBP-1 and then esterified into TG [32,48,49]. Together, these studies, coupled with decreased Cer levels and increased epidermal water loss in IL-4 treated epidermis equivalent [50], suggest that ingested CJLP55 modulates adaptive immunity cytokine levels (more likely to suppress Th-2 cytokine levels) systemically via altered bacterial EV phylum flora, which may lower TG of sebum and improve skin hydration with increased Cer levels, thereby decreasing follicular plugging and inflammation from *C. acnes* bacteria in acne lesions. Future studies with a larger number of acne patients would facilitate evaluation of systemic alterations in adaptive immunity cytokines as well as in insulin, IFG-1 and BCAA levels and intestinal and skin bacterial flora after *L. plantarum* CJLP55 supplement intervention.

## 5. Conclusions

This randomized, double-bind, placebo-controlled study demonstrated that a 12-week supplementation of CJLP55 decreased the clinical severity of acne vulgaris, *Proteobacteria* proportion in urine EV phylum, and TG of sebum. The last feature was inversely correlated to increases in the *Firmicutes* proportion of urine EV phylum, Cer, and hydration of skin. CJLP55 supplement was beneficial to clinical state, skin sebum, and hydration and urine bacterial EV phylum flora in patients with acne vulgaris. Dietary *Lactobacillus plantarum* CJLP55 may be a potential alternative therapy or may serve as an adjunct to conventional therapies for the treatment of acne vulgaris.

## Figures and Tables

**Table 1:** Baseline characteristics by groups.

Characteristics	CJLP55 Group	Placebo Group	*p* Value
	(*n* = 14)	(*n* = 14)	
Sex (male/female)	7/7	5/9	0.445 ^2^
Age (years)	24.29 ± 0.73 ^1^	23.86 ± 0.80	0.695 ^3^
Body mass index (kg/m^2^)	20.74 ± 0.64	21.39 ± 0.55	0.401 ^3^
Inflammatory lesion count (ILC)	17.79 ± 3.19	19.64 ± 3.81	0.874 ^3^
Total lesion count (TLC)	72.93 ± 10.11	98.14 ± 15.75	0.189 ^3^
Acne grade	3.21 ± 0.19	3.14 ± 0.14	0.734 ^3^

^1^ Mean ± SEM (all such values). ^2^ *p* value for difference in observed numbers of men and women from theoretical frequencies between the CJLP55 and placebo groups by chi-square test. ^3^ *p* value for difference between the CJLP55 and placebo groups by Student’s *t* test (age, TLC) or Mann–Whitney U test (body mass index, ILC, acne grade).

**Table 2:** Altered acne lesion count and acne grade by groups.

	CJLP55 Group (*n* = 14, 7 Male/7 female)			Placebo Group (*n* = 14, 5 Male/9 Female)			% Change between Groups (95%CI)	*p* Value ^4^
	Baseline	Week12	% Change	Baseline	Week12	% Change		
**Inflammatory lesion count (ILC)**								
Male subjects	24.86 ± 4.74	6.71 ± 1.36 **^1,2^	−72.99 ± 14.86	20.40 ± 6.78	15.40 ± 7.36	−24.51 ± 15.88	−48.48 (−97.84 to 0.89) ^3^	0.054
Female subjects	10.71 ± 2.24	4.57 ± 1.54 *^2^	−57.33 ± 19.79	19.22 ± 4.89	14.00 ± 2.13	−27.17 ± 16.31	−30.17 (−84.67 to 24.34)	0.255
All subjects	17.79 ± 3.19	5.64 ± 1.03 ***^2^	−68.27 ± 14.83	19.64 ± 3.81	14.50 ± 2.79 *^2^	−26.18 ± 11.43	−42.09 (−80.57 to −3.61)	0.033
**Total lesion count (TLC)**								
Male subjects	98.00 ± 13.73	39.57 ± 7.00 **^2^	−59.62 ± 9.48	95.40 ± 28.62	78.00 ± 20.52	−18.24 ± 10.83	−41.38 (−73.67 to −9.10)	0.017
Female subjects	47.86 ± 6.67	27.57 ± 6.15 **^2^	−42.39 ± 10.41	99.67 ± 19.97	102.78 ± 20.02	3.12 ± 18.41	−45.51 (−94.67 to 3.65)	0.031
All subjects	72.93 ± 10.11	33.57 ± 4.77 ***^2^	−53.97 ± 10.04	98.14 ± 15.75	93.93 ± 14.69	−4.29 ± 12.58	−49.67 (−82.76 to −16.59)	0.002
**Acne grade**								
Male subjects	3.71 ± 0.18	2.86 ± 0.26 *^2^	−23.08 ± 7.02	3.20 ± 0.20	3.40 ± 0.24	6.25 ± 6.25	−29.33 (−51.36 to −7.29)	0.030
Female subjects	2.71 ± 0.18	2.00 ± 0.31 *^2^	−26.31 ± 6.79	3.11 ± 0.20	3.22 ± 0.15	3.57 ± 8.37	−29.89 (−54.04 to −5.74)	0.012
All subjects	3.21 ± 0.19	2.43 ± 0.23 **^2^	−24.44 ± 4.81	3.14 ± 0.14	3.29 ± 0.13	4.55 ± 5.64	−28.99 (−44.23 to −13.75)	0.009

CI, confidence interval. ^1^ Mean ± SEM for all such values. ^2^ *p* value for difference from baseline within CJLP55 or placebo groups by paired *t* test (total lesion count) or Wilcoxon signed rank test (inflammatory lesion count, acne grade) (* *p* < 0.05, ** *p* < 0.01, *** *p* < 0.001). ^3^ Mean (95% CI) for all such values. ^4^ *p* value for difference of percent change between the CJLP55 and placebo groups by Student’s *t* test (inflammatory lesion count) or Mann–Whitney U test (total lesion count, acne grade).

**Table 3:** Altered skin sebum, hydration, and pH by groups.

	CJLP55 Group (*n* = 14, 7 Male/7 Female)			Placebo Group (*n* = 14, 5 Male/9 Female)			% Change between Groups (95%CI)	*p* Value ^4^
	Baseline	Week12	% Change	Baseline	Week12	% Change		
**Skin sebum (μg/cm^2^)**								
All subjects	178.82 ± 12.98	147.11 ± 16.40 *^1,2^	−17.74 ± 6.04	202.36 ± 15.28	199.39 ± 16.44	−1.47 ± 4.92	−16.27 (−32.28 to −0.26) ^3^	0.005
**Skin hydration (capacitance in au.)**								
All subjects	73.47 ± 1.53	77.64 ± 1.99	5.67 ± 2.95	72.67 ± 1.73	66.24 ± 1.78	−8.85 ± 2.58	14.52 (6.47 to 22.57)	0.003
**Skin pH**								
All subjects	6.67 ± 0.15	5.40 ± 0.10	−18.93 ± 2.70	6.69 ± 0.11	5.61 ± 0.11	−16.17 ± 2.42	−2.76 (−10.21 to 4.69)	0.453

CI, confidence interval; au, arbitrary unit. ^1^ Mean ± SEM for all such values. ^2^ *p* value for difference from baseline within CJLP55 or placebo groups by paired *t* test (skin sebum, skin hydration) or Wilcoxon signed rank test (skin pH) (* *p* < 0.05). ^3^ Mean (95% CI) for all such values. ^4^ *p* value for difference of percent change between the CJLP55 and placebo groups by Student’s *t* test (skin pH) or Mann–Whitney U test (skin sebum, skin hydration).

## Data Availability

The data presented in this study are available in Supplementary Materials (File S2: Raw research data).

## Supplementary Materials

The following are available online at https://www.mdpi.com/article/10.3390/nu13041368/s1. Figure S1: Consort flow diagram. File S1: Raw research data. File S2: Consort 2010 checklist.
