# Supplementary figures and images for: Effects of Lactobacillus plantarum CJLP55 on Clinical Improvement, Skin Condition and Urine Bacterial Extracellular Vesicles in Patients with Acne Vulgaris: A Randomized, Double-Blind, Placebo-Controlled Study
Source: Nutrients. 2021 Apr 19;13(4):1368. doi: 10.3390/nu13041368 (PMC8073324; doi:10.3390/nu13041368)

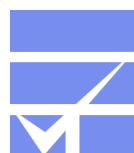

# CONSORT

TRANSPARENT REPORTING of TRIALS

## CONSORT 2010 Flow Diagram

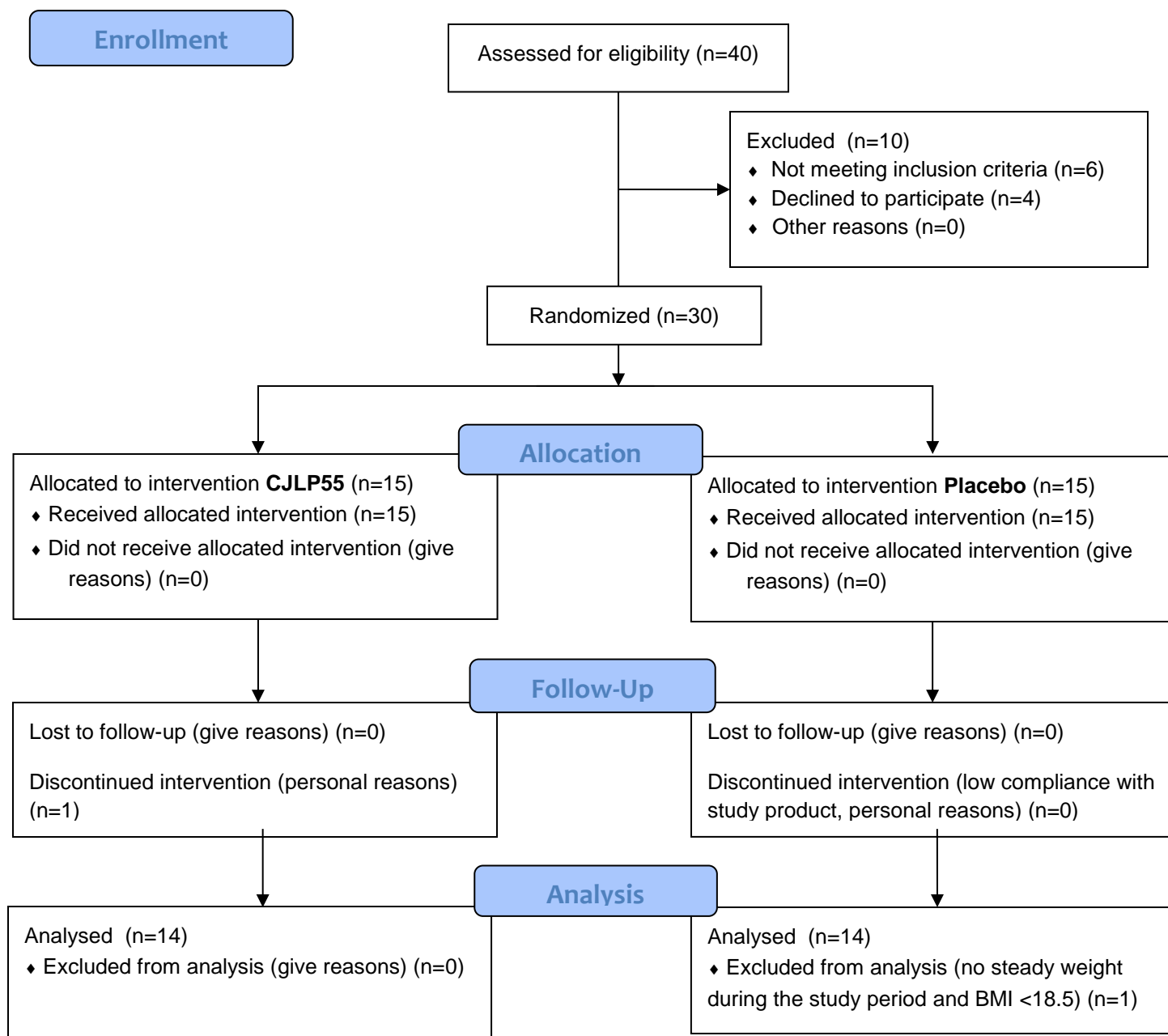

Supplement: Supplementary file 1 [file nutrients-13-01368-s001.zip › nutrients-1183285-supplementary/YCHOFigureS1CONSORT2010FlowDiagram2021NutrientswithFormat.pdf]
